# Respiratory pattern of diaphragmatic breathing and pilates breathing in COPD subjects

**DOI:** 10.1590/bjpt-rbf.2014.0042

**Published:** 2014

**Authors:** Karina M. Cancelliero-Gaiad, Daniela Ike, Camila B. F. Pantoni, Audrey Borghi-Silva, Dirceu Costa

**Affiliations:** 1 Programa de Pós-Graduação em Fisioterapia, Universidade Federal de São Carlos (UFSCar), São Carlos, SP, Brazil; 2 Programa de Pós-Graduação em Ciências da Reabilitação, Universidade Nove de Julho (UNINOVE), São Paulo, SP, Brazil

**Keywords:** physical therapy, COPD, plethysmography, breathing

## Abstract

**BACKGROUND:**

Diaphragmatic breathing (DB) is widely used in pulmonary rehabilitation (PR) of patients with chronic obstructive pulmonary disease (COPD), however it has been little studied in the scientific literature. The Pilates breathing (PB) method has also been used in the rehabilitation area and has been little studied in the scientific literature and in COPD.

**OBJECTIVES:**

To compare ventilatory parameters during DB and PB in COPD patients and healthy adults.

**METHOD:**

Fifteen COPD patients (COPD group) and fifteen healthy patients (healthy group) performed three types of respiration: natural breathing (NB), DB, and PB, with the respiratory pattern being analyzed by respiratory inductive plethysmography. The parameters of time, volume, and thoracoabdominal coordination were evaluated. After the Shapiro-Wilk normality test, ANOVA was applied followed by Tukey's test (intragroup analysis) and Student's t-test (intergroup analysis; p<0.05).

**RESULTS:**

DB promoted increase in respiratory volumes, times, and SpO_2_ as well as decrease in respiratory rate in both groups. PB increased respiratory volumes in healthy group, with no additional benefits of respiratory pattern in the COPD group. With respect to thoracoabdominal coordination, both groups presented higher asynchrony during DB, with a greater increase in the healthy group.

**CONCLUSIONS:**

DB showed positive effects such as increase in lung volumes, respiratory motion, and SpO_2_ and reduction in respiratory rate. Although there were no changes in volume and time measurements during PB in COPD, this breathing pattern increased volumes in the healthy subjects and increased oxygenation in both groups. In this context, the acute benefits of DB are emphasized as a supporting treatment in respiratory rehabilitation programs.

## Introduction

Diaphragmatic breathing (DB) is widely used in pulmonary rehabilitation in patients with chronic obstructive pulmonary disease (COPD). The main objectives are to improve abdominal movement and at the same time reduce the time of thoracic excursion and the activity of the respiratory muscles of the ribcage^1^^,^ 2. Some of the beneficial effects of DB are the improvement in maximum exercise tolerance^3^, blood gases (increase in partial oxygen pressure and reduction in partial carbon dioxide pressure)^1^, and in diaphragm muscle mobility^2^.

The Pilates breathing (PB) method is another frequently used type of respiration that differs from DB. PB requires deep breathing while keeping the abdomen pulled in by means of active contraction of the transverse abdominal (TrA) and pelvic floor muscles^4^. Although the Pilates method is growing in both the area of fitness and rehabilitation, there is scarcely any scientific research on the subject, particularly in the area related to respiration. Thus, better knowledge of the specific breathing technique of this method is necessary, particularly when applied to individuals with diseases such as COPD, who present diaphragmatic muscle dysfunction^2^.

Therefore, we hypothesized that the respiratory patterns during DB and PB are different because in DB there is a diaphragmatic excursion with abdominal projections and in PB the abdomen is contracted and chest breathing is encouraged, and we conducted the present study in order to investigate the different respiratory patterns induced by the two techniques. In this context, the aim of the present study was to compare these respiratory patterns in COPD patients and healthy adults, evaluated by the respiratory inductance plethysmography (RIP) method.

## Method

### Design and study population

This was a prospective, randomized, and crossover trial. To establish the COPD population, a total of 30 subjects of both genders were screened, including patients with stable and moderate to severe COPD^5^, among whom fifteen subjects (8 men and 7 women) were selected for inclusion in the study. The selected subjects had a documented medical history of COPD, were receiving medical therapy with pulmonary drugs, were smokers or former smokers, and none had any clinical or physiological features of bronchial asthma. The exclusion criteria were age over 80 years, obesity, history of recent exacerbation, uncontrolled arterial hypertension, and need for home oxygen therapy.

For the healthy group, 15 subjects were also included according to these criteria: healthy men and women aged between 40 and 80 years. The exclusion criteria were obesity, presence of pulmonary, cardiovascular, neurological, and orthopedic diseases, or any other dysfunction that hindered the participation in the study. In this group there was no sample loss.

The study was approved by the Research Ethics Committee of Universidade Federal de São Carlos (UFSCar), São Carlos, SP, Brazil (protocol 073/2009). All the subjects signed an informed consent form to participate in the research.

### Measurements

The measurements that were studied were taken on two different days. On the first day, the subjects underwent a clinical assessment, and baseline characteristics, such as age, gender, weight, height, body mass index (BMI), were recorded. Respiratory muscle strength represented by maximal inspiratory pressure (MIP) and maximal expiratory pressure (MEP) were assessed with an analog vacuum manometer (Ger-Ar^®^, São Paulo, SP, Brazil) in accordance with the recommendation in the literature^6^. Spirometry was performed with a portable spirometer (Easy One^®^, Andover, MA, USA) to obtain forced vital capacity (FVC); forced expiratory volume in 1 second (FEV_1_); and the FEV1/FVC ratio. The procedure was performed in accordance with the guidelines of the American Thoracic Society^7^.

**Figure 1:**
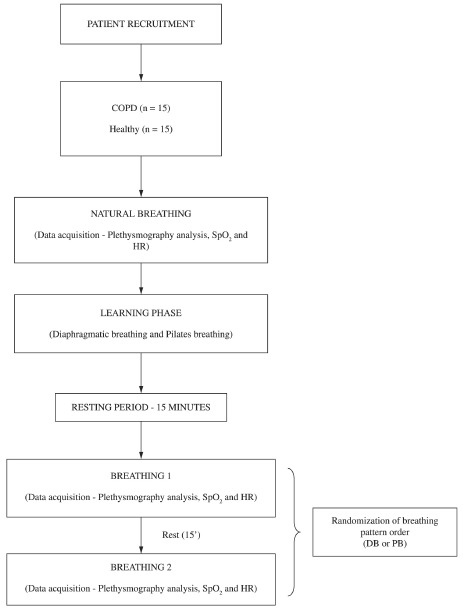
Experimental protocol.

On the second day, the subject returned for the experimental procedure. Initially, for the baseline measure, the respiratory pattern was recorded for two minutes during NB. After this, the physical therapist taught the participants the DB and PB techniques (learning phase). Next, these patients were asked to perform each technique in turn to retain their effectiveness. After the learning phase and a period of 15 minutes rest, the respiratory pattern was recorded for 2 minutes during DB and PB performed in a randomly assigned order, which was contained in opaque, sealed envelopes that were shuffled, distributed, and opened immediately before the evaluation. The breathing techniques were performed in the supine position^2^ with a 15-minute interval. The inspiratory and expiratory times were not standardized, and the subjects were free to perform the exercises at their own pace (Figure 1). All subjects completed both breathing techniques and care was taken to ensure that the proportion of subjects who started assessing DB was equal to that of PB.

## Experimental procedures

In the present study, the measurements were investigated at baseline (NB) and during two types of respiration as follows:

**BASELINE/NATURAL BREATHING (NB) - **the patients were placed in the supine position and were instructed to remain relaxed during the specified time. In this period, no breathing frequency was induced to allow the detection of each patient's breathing pattern.

**DIAPHRAGMATIC BREATHING (DB) - **the patient lay in the supine position with one hand placed at the top of the abdominal area and the other, at the top of the upper thorax. The emphasis was on outward abdominal movement during inspiration and inward abdominal movement during expiration^2^^,^ 8.

**PILATES BREATHING (PB) - **PB was performed according to the recommendations of Menezes^9^: "Keep the neck and shoulders relaxed; Allow the respiration to flow: do not hold your breath at any point; Breathe through your nose without allowing your shoulders to lift; Without stopping, breathe out through your mouth with a sigh; Breathing out through your teeth, with your lips pursed". In addition to deep breathing, the abdomen had to be kept pulled in by active contraction of the TrA and pelvic floor muscles^4^.

### Respiratory Pattern measurements

The respiratory pattern was assessed by respiratory inductive plethysmography (RIP) using the LifeShirt System (Vivometrics Inc., Ventura, CA, USA) and was monitored using the thoracic and abdominal inductance plethysmography bands integrated in the LifeShirt positioned at the level of the nipples and umbilicus, respectively. Data were recorded with a portable device and stored in a flash memory card inserted in the LifeShirt recorder, then downloaded to a computer and analyzed with the VivoLogic analysis software program (Vivometrics Inc., Ventura CA, USA) that accompanies the LifeShirt.

For the volumetric adjustment procedure, the participants were asked to wear a nose clip and breathe in and out 7 times through a mouthpiece attached to an 800-ml plastic bag, filling and emptying the bag completely with each breath. This procedure was conducted in the sitting and standing posture after appropriate pauses, twice for each posture, and repeated if participants did not adhere to instructions, until it was successfully performed.

In the respiratory pattern analyses, the following variables were used:

- Volume measures: Inspiratory tidal volume (ViVol); expiratory tidal volume (VeVol), and minute ventilation (Vent)
- Time measures: Respiratory Rate (Br/M); inspiratory time (Ti); expiratory time (Te), and total breath time (Tt)
- Thoracoabdominal coordination measures: Percent Rib Cage Inspiratory Contribution to Tidal Volume Ratio (%RCi); Labored Breathing Index (LBI); phase relation during inspiration (PhRIB); phase relation during expiration (PhREB); phase relation of the entire breath (PhRTB); and phase angle (PhAng)

To obtain the RIP sum signal for absolute volume in ml, a quantitative calibration was carried out before the analysis of respiratory variables. Breath-by-breath analysis was performed during a 2-minute period and converted into mean values for later comparisons by statistical analysis.

### Peripheral oxygen saturation measurements

During the breathing exercises, peripheral oxygen saturation measurements (SpO_2_) and heart rate (HR) were determined by pulse oximetry (Nonim^®^ 8500A, Plymouth, MN, USA).

### Statistical analysis

The Shapiro-Wilk test was applied to establish data frequency distribution and as data presented normality, repeated measures ANOVA with Tukey's post-Hoc (intragroup analyses) and unpaired Student's t-test (intergroup analysis) were performed. A *p*-value of 0.05 was considered statistically significant. The Prism 3.0^®^ software program was used. The power analysis was performed using GraphPad StatMate^®^ 2.00 and the statistical power was 99%.

## Results

The characteristics of the healthy group and the COPD group are shown in Table 1.

**Table 1:** Anthropometric variables and spirometric variables of the healthy group and COPD group (n=15/group).

Variable	Healthy	COPD	P
Age (years)	62.5±9.4	65.3±7.3	0.37
Gender (M/F)	7/8	8/7	1.00
Height (m)	1.70±0.05	1.65±0.11	0.11
Weight (kg)	70.1±8.1	66.3±10.9	0.29
BMI (kg/m^2^)	24.2±2.3	24.6±4.8	0.78
FVC (% predicted)	102.6±10.4	70.2±16.2	<0.0001
FEV_1_ (% predicted)	102.8±10.6	46.9±11.1	<0.0001
FEV_1_/FVC (% predicted)	98.4±6.1	68.6±11.0	<0.0001
MIP (cmH_2_O)	–84.7±29.8	–64.7±27.2	0.07
MEP (cmH_2_O)	104.7±35.8	81.4±28.6	0.06

BMI= body mass index; FVC= forced vital capacity; FEV_1_= forced expiratory volume in 1 second; FEV_1_/FVC: Tiffeneau index; MIP: maximal inspiratory pressure; MEP: maximal expiratory pressure. Values are mean±SD

In the COPD group, 9 individuals were former smokers and 6 were smokers. As regards the classification of COPD, 6 presented with a moderate obstruction and 9 with severe obstruction. As expected, the healthy group presented higher values for pulmonary variables compared with the COPD group.

Considering the respiratory pattern in the intragroup analysis, only DB differed from NB in the COPD group, with an increase in volume measures (ViVol: 121%; VeVol: 120%; Vent: 63%), Ti (46%), Te (55%), and Tt (52%) and a decrease in Br/M (34%). In addition, DB induced an increase in the thoracoabdominal coordination measures (PhRIB: 187%; PhREB: 167%; PhRTB: 178%; PhAng: 178%) when compared with NB. SpO_2_ increased in both DB (4.2%) and PB (4.1%) compared with NB (Table 2). In contrast, HR did not differ between the three respirations (NB:74±10; DB:75±10; PB:79±10 bpm). When comparing PB with DB, DB showed higher values for ViVol (49%), VeVol (65%), Ti (35%), Te (67%), Tt (56%), PhRIB (81%), PhREB (71%), PhRTB (68%), and PhAng (119%) and lower values for Br/M (35%; Table 2).

**Table 2:** Plethysmography analysis measures and peripheral oxygen saturation (SpO2) of the COPD group (A) and the healthy group (B) (n=15/group).

A	NB	DB	PB
*Volume measures*			
ViVol	397.9±125.3	880.5±421.4 *	591.4±377.5 # †
VeVol	400.9±128.7	881.7±426.4 *	533.5±291.3 # †
Vent	6.0±2.4	9.8±2.5 *	8.9±4.3
*Time measures*			
Br/M	16.7±3.8	11.0±3.5 *	16.9±7.4 #
Ti	1.3±0.3	1.9±0.4 * †	1.4±0.3 # †
Te	2.9±1.3	4.5±2.0 *	2.7±0.9 #
Tt	4.2±1.5	6.4±2.1 *	4.1±1.1 #
Ti/Tt	0.34±0.06	0.33±0.09	0.36±0.07 †
*Thoracoabdominal coordination measures*			
%RCi	54.5±28.1	50.6±48.4	61.1±28.2
LBI	1.07±0.26	1.18±0.26	1.03±0.03
PhRIB	13.5±12.9 †	38.8±21.6 *	21.4±10.5 #
PhREB	13.9±8.0 †	37.1±19.0 *	21.7±9.8 #
PhRTB	13.4±8.0 †	37.2±19.6 *	22.1±9.5 #
PhAng	24.1±22.1 †	67.0±47.7 * †	30.6±12.3 # †
Oximetry			
SpO_2_	95.4±3.4	99.4±1.4 *	99.3±1.6 *
** B**	**NB**	**DB**	**PB**
*Volume measures*			
ViVol	361.9±145.4	1347.8±524.3 *	948.6±439.3 * #
VeVol	368.3±145.2	1420.5±584.3 *	993.0±457.9 * #
Vent	5.6±1.8	13.6±5.6 *	14.4±4.7 *
*Time measures*			
Br/M	16.4±3.7	11.8±4.8 *	16.2±3.4 #
Ti	1.4±0.4	2.9±0.9 *	1.8±0.4 #
Te	2.3±0.5	5.2±1.8 *	2.5±0.6 #
Tt	3.7±0.8	8.1±2.5 *	4.2±0.9 #
Ti/Tt	0.39±0.04	0.38±0.07	0.43±0.04 #
*Thoracoabdominal coordination measures*			
%RCi	63.3±16.3	66.7±15.5	80.9±18.3 *
LBI	1.00±0.00	1.08±0.06	1.05±0.08
PhRIB	5.7±3.0	29.6±14.6 *	25.8±12.3 *
PhREB	5.8±2.7	30.7±14.2 *	28.0±10.1 *
PhRTB	5.7±2.5	26.8±12.7 *	26.2±10.4 *
PhAng	9.1±4.2	39.1±19.1 *	21.1±9.5 * #
Oximetry			
SpO_2_	97.4±1.6	99.7±0.7 *	99.5±0.8 *

NB= natural breathing; DB= diaphragmatic breathing; PB= pilates breathing; ViVol= inspiratory tidal volume; VeVol= expiratory tidal volume; Vent= minute ventilation; Br/M= Respiratory Rate; Ti= Inspiratory Time; Te= Expiratory Time; Tt= Total Breath Time; Ti/Tt= fractional inspiratory time; %RCi= Percent Rib Cage Inspiratory Contribution to Tidal Volume Ratio; LBI= Labored Breathing Index; PhRIB= phase relation during inspiration; PhREB= phase relation during expiration; PhRTB= phase relation of entire breath; PhAng= phase angle (PhAng), SpO_2_= peripheral oxygen saturation. Values are mean±SD. Intragroup analysis: *<0.05 compared with NB. #<0.05 compared with DB (ANOVA). Intergroup analysis: † compared with healthy group in the same breathing (unpaired Student's t test)

Similarly, in the healthy group, DB induced an increase in volume measures (ViVol: 272%, VeVol: 286%, Vent: 143%), an increase in Ti (107%), Te (126%), and Tt (119%) and a decrease in Br/M (28%) when compared with NB. DB also showed higher values for thoracoabdominal coordination variables when compared with NB (PhRIB: 419%; PhREB: 429%; PhRTB: 370%, PhAng: 330%). PB presented an increase in volume (ViVol: 162%; VeVol: 170%; Vent: 157%) and thoracoabdominal coordination values (PhRIB: 353%; PhREB: 383%; PhRTB: 360%; PhAng: 132%; %RCi: 28%) when compared with NB. Similarly to the COPD group, SpO_2_ increased in both DB (2.4%) and PB (2.2%) compared with NB (Table 2). When comparing PB with DB, the latter showed higher values for ViVol (42%), VeVol (43%), Ti (61%), Te (108%), Tt (93%), and PhAng (85%) and lower values for Br/M (27%) and Ti/Tt (12%; Table 2). HR did not differ between the three breathing patterns (NB:69±4; DB:70±5; PB:70±5bpm).

In the intergroup analysis, the COPD group showed lower values for ViVol (37%), VeVol (46%), Ti (22%), and Ti/Tt (16%) and higher values for PhAng (45%) during PB, with lower values for Ti (35%) and higher values for PhAng (71%) during DB. In NB, the COPD group showed higher values for PhRIB (137%), PhREB (140%), PhRTB (135%), and PhAng (165%). With regard to SpO_2_, there was no difference between the groups during any of the breathing patterns. HR was higher in the PB of the COPD group (79±10bpm) when compared with the healthy group (70±5bpm, p=0.005).

## Discussion

The main results of this study showed that DB favored greater respiratory volumes and times in both groups, contributing to the reduction in Br/M and increase in SpO_2_, compared with NB. PB was able to increase respiratory volumes in the healthy group, compared with NB, with no additional benefits in the respiratory pattern of the COPD group. With respect to thoracoabdominal coordination, as expected, both groups presented higher asynchrony during DB, compared with NB, with a greater increase in the healthy group. These results are important, since they may co-substantiate the potential beneficial effects of these respiratory breathing modalities in COPD patients in contrast with healthy subjects.

It is known that diaphragmatic dysfunction is an important deleterious consequence of the progression of the severity of COPD. With the increase in air flow resistance, air trapping, and hyperinflation in this disease, the inspiratory muscles are passively shortened and placed at a mechanical disadvantage^10^^,^ 11. Therefore, a progressive reduction occurs in the mobility of the diaphragm and in its relative contribution to thoracoabdominal movement^12^^-^ 14, and as a compensatory mechanism, there is greater recruitment of the respiratory muscles of the rib cage^15^^,^ 16. In this context, both the reduction in diaphragm mobility and the greater activity of the rib cage respiratory muscles are associated with the increase in dyspnea and intolerance to physical exercise^17^^-^ 19.

To reduce or minimize these alterations, studies have been conducted with DB as a form of therapy for improving diaphragmatic mobility and thereby reducing the deleterious effects of diaphragmatic dysfunction. According to the ATS^20^, DB is a respiratory strategy frequently taught as a component of self-treatment in COPD patients, with the goal of minimizing the respiratory demand of the disease and reducing its impact on daily life. In the study by Yamaguti et al.^2^, a DB training program in COPD patients promoted improvement in diaphragm mobility, with an increase in the participation of the diaphragm during natural respiration, resulting in an improvement in functional capacity, in addition to improvement in health-related quality of life. Other studies^1^^,^ 21 found an improvement in gas exchange in the respiratory patterns^22^^,^ 23 and in oxygen consumption^24^.

It has been suggested that the beneficial effects of DB depend on the COPD patients' characteristics, such as severity of the disease, pulmonary hyperinflation, and adequate diaphragmatic movement, an essential condition for the success of the respiratory technique^8^. Moreover, a paradoxal abdominal respiratory pattern and worsening of dyspnea and fatigue during the technique are criteria for modifying or interrupting DB^8^. In this context, it is important to mention that the subjects of the present study adequately performed DB, which was monitored by the physical therapist, without any report of dyspnea. DB was beneficial to the COPD patients because it promoted a reduction in respiratory rate and increased the lung volumes, which is in agreement with the proposal of Cahalin et al.^8^.

DB is frequently applied in pulmonary rehabilitation programs, and its efficacy in improving pulmonary volumes and SpO_2_ and reducing Br/M has been documented^18^^,^ 25. In the present study, the beneficial effects of DB on respiratory volumes and times and oxygenation in both groups were also observed when compared with NB. An important issue to consider as regards DB is the thoracoabdominal coordination during the technique, which was shown to be increased^26^. Therefore, the benefits of the technique could be questioned, particularly in COPD patients, who already present higher asynchrony in comparison with healthy subjects.

To clarify this issue, the thoracoabdominal measures were evaluated during the technique, comparing the COPD patients' results with those of healthy-matched subjects. A similar response was found in both groups, with an increase in asynchrony values in comparison with those of NB. Moreover, the healthy subjects presented a higher increase in all asynchrony measures. As expected of a respiratory technique that emphasizes greater use of the diaphragm and abdominal breathing components thus generating "asynchrony" during the respiratory cycle, both groups presented a similar respiratory pattern behavior. In this study, asynchrony between the thoracic and abdominal compartments was evaluated by PhAng^23^, and when the rib cage and abdomen move in perfect synchrony, the PhAng is 0º. However, with the increase in thoracoabdominal asynchrony, this value is close to 180º. In this context, although DB increased the PhAng, it maintained mean values of 70° and did not attain maximum asynchrony values. For this reason, the changes in the measurements related to synchronism cannot be interpreted as an increase in asynchronism, since the increase in mean values remained below 70°.

The increase in thoracoabdominal asynchrony during DB is possibly related to the greater use of the diaphragm. This has also been reported in healthy subjects^25^ and other respiratory exercises^26^ as mentioned above. It is important to emphasize that DB was performed with inward abdominal movement during expiration. This action can improve the next inspiration since it provides a better mechanical positioning of the diaphragm.

In the present study, although the subjective perceived exertion scale was not used, the SpO_2_ was elevated and none of the patients reported dyspnea when breathing correctly and during the proposed time.

The PB technique differs to a great extent from that of DB. To perform the exercises of the Pilates^®^ method, it is necessary to breathe deeply, maintaining the abdomen contracted by active contraction of the local and overall stabilizing muscles of the lumbar spine, in addition to the diaphragm muscle and the pelvic floor muscles^9^. According to Barr et al.^27^, the diaphragm muscle works as the roof of a cylinder of muscles that surround the spine and assist with stability. It is one of the main contributors towards maintaining intra-abdominal pressure and preventing displacement of the viscera by contraction, mainly of the TrA muscle.

The specific respiration of the Pilates method is known as lateral breathing, which avoids expansion of the abdomen with the aim of using the thoracic and ribcage muscles to generate lateral expansion of the ribcage, increasing the space for the lungs to expand and avoiding the movement of the abdomen so as not to leave the lumbar region unprotected^9^^,^ 28. Thus, it is clear that the objectives of the breathing techniques differ and that the diaphragm muscle in PB also acts as a stabilizer of the lumbar spine. Therefore, as respiration is a little restricted because no movement occurs in the abdominal compartment, the results of this study showed that in the COPD group there were no changes, for example, in the pulmonary volumes, unlike DB, in which there is a diaphragmatic excursion with abdominal projection. This more restricted respiratory movement in PB did not promote alteration in any respiratory patterns evaluated in the individuals with COPD in the present study. In the healthy group, however, PB promoted alterations such as an increase in lung volumes, %RCi, and SpO_2_. %RCi has been described as a measure that represents the percent contribution of the rib cage excursions to the tidal volume. Thus, because the movement of the rib cage is greater in the healthy subjects, this was probably detected only in the healthy group. This fact may also explain the alterations in other measures, for example, lung volume and synchronism, due to the fact that the ribcage of the healthy group showed no rigidity and thus the movements were greater.

It should be pointed out that PB specifically promoted a breathing pattern with greater thoracic expansibility. In individuals with COPD, this respiratory pattern may have been influenced by the disease because there is the presence of thoracic rigidity and diminished expansibility, which may also explain the absence of increases in pulmonary volumes, respiratory times, and even in thoracoabdominal asynchrony. However, the active contraction of the TrA muscle can bring long-term benefits, since it provides stabilization of the abdominal compartment and supports the descent of the diaphragm.

With regard to SpO_2_, the results of the present study showed that there was an increase during PB in both groups, which may be due to the use of pursed-lip breathing. According to some authors^29^, this type of breathing is associated with a partial increase in oxygen pressure in the arterial blood and SpO_2_.

With regard to the limitations of this study, one is that the SpO_2 _measurements were made using a system that did not store memory as in the plethysmography measurements. Other limitations were the absence of a COPD control group and the sample size.

In view of the foregoing discussion, DB showed positive effects such as an increase in lung volumes, respiratory motion, SpO_2_, and reduction in respiratory rate. Although there were no changes in the volume and time measurements during PB in COPD, this breathing pattern increased volumes in the healthy subjects and increased oxygenation in both groups. In this context, the acute benefits of DB are emphasized as a supporting treatment in respiratory rehabilitation programs. Future studies should focus on the effects of both respiratory patterns in other outcomes in order to confirm the positive or negative effects of these interventions.
